# ASV portal: an interface to DNA-based biodiversity data in the Living Atlas

**DOI:** 10.1186/s12859-022-05120-z

**Published:** 2023-01-05

**Authors:** Maria Prager, Daniel Lundin, Fredrik Ronquist, Anders F. Andersson

**Affiliations:** 1 Science for Life Laboratory, Department of Ecology, Environment and Plant Sciences, Stockholm University, 106 91 Stockholm, Sweden; 2 Department of Microbiology, Tumor and Cell Biology, Karolinska Institutet, 171 77 Stockholm, Sweden; 3 Centre for Ecology and Evolution in Microbial Model Systems, Linnaeus University, 391 82 Kalmar, Sweden; 4 Department of Bioinformatics and Genetics, Swedish Museum of Natural History, P.O. Box 50007, 104 05 Stockholm, Sweden; 5 Science for Life Laboratory, Department of Gene Technology, KTH Royal Institute of Technology, 171 21 Stockholm, Sweden

**Keywords:** Biodiversity informatics, Species occurrence, Darwin core, Amplicon sequencing, Metabarcoding, eDNA, BLAST

## Abstract

**Background:**

The Living Atlas is an open source platform used to collect, visualise and analyse biodiversity data from multiple sources, and serves as the national biodiversity data hub in many countries. Although powerful, the Living Atlas has had limited functionality for species occurrence data derived from DNA sequences. As a step toward integrating this fast-growing data source into the platform, we developed the Amplicon Sequence Variant (ASV) portal: a web interface to sequence-based biodiversity observations in the Living Atlas.

**Results:**

The ASV portal allows data providers to submit denoised metabarcoding output to the Living Atlas platform via an intermediary ASV database. It also enables users to search for existing ASVs and associated Living Atlas records using the Basic Local Alignment Search Tool, or via filters on taxonomy and sequencing details. The ASV portal is a Python-Flask/jQuery web interface, implemented as a multi-container docker service, and is an integral part of the Swedish Biodiversity Data Infrastructure.

**Conclusion:**

The ASV portal is a web interface that effectively integrates biodiversity data derived from DNA sequences into the Living Atlas platform.

## Background

Biodiversity research is rapidly developing into big-data science, enabling researchers to model processes that affect entire biotas and to predict ecosystem-wide effects of environmental change. To facilitate this, infrastructures that provide open access to species observation data for all types of life are crucial. The Living Atlas (LA) is an infrastructure for integration of biodiversity data from multiple sources with environmental and contextual information. It was originally developed by the Atlas of Living Australia, in response to growing demands of the biodiversity research community for open access to extensive databases and analysis tools [1]. It is, however, also supported by the Global Biodiversity Information Facility [2], and now serves as the main biodiversity data hub in 27 countries and regions [3]. The software is developed in open collaboration, and more than 100 developers have contributed to the codebase.

Although the LA accommodates less traditional data types such as images, or output from animal tracking devices, it has so far offered limited functionality for DNA sequence-based observations. Meanwhile, molecular methods for species observation, in particular metabarcoding (amplicon sequencing of taxonomic marker genes) of environmental DNA (eDNA) and bulk samples, are becoming increasingly important tools for documenting the diversity of life [4], especially in the microscopic realm (prokaryotes, protists and fungi; see e.g., [5]).

We identified three features that would make the LA platform more useful for handling occurrence data derived from metabarcoding: (1) the option to store processed barcode sequences in the form of Amplicon Sequence Variants (ASVs), underlying occurrences in the atlas, and to use the Basic Local Alignment Search Tool (BLAST; [6]) to find such occurrences, (2) the possibility of searching for ASVs and occurrence records based on sequencing details, such as target genes and primers, and (3) a dynamic approach to taxonomic annotation of observed ASVs, allowing for easy updates as reference databases develop. Below, we present an application that provides these features, and functions as a semi-integrated LA module.

### Implementation

**Fig. 1:**
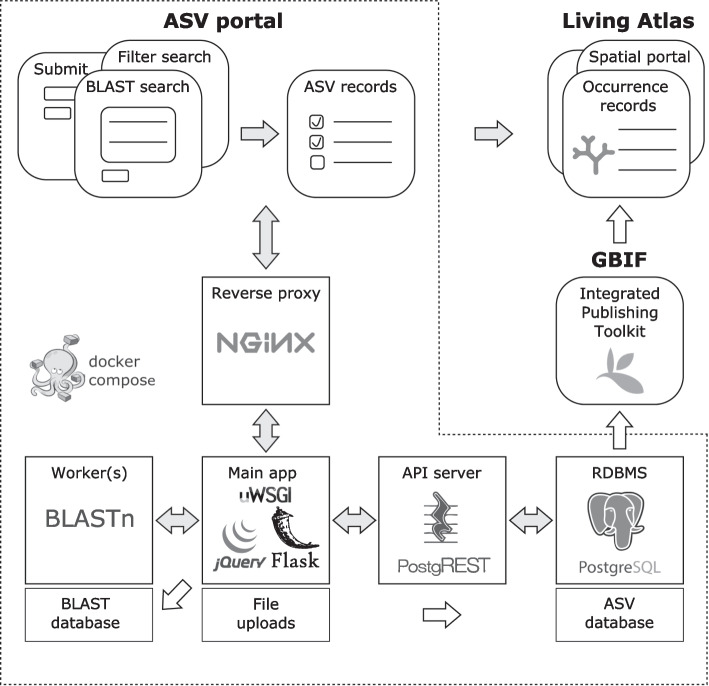
ASV portal components and connections to biodiversity data platforms. Illustrated parts include Docker services (squares) and volumes (rectangles), web pages (squircles), general data and user flow (grey arrows), as well as administrator interactions (white arrows)

The ASV portal is a web interface to sequence-based biodiversity observations in the LA platform, and is implemented as five separate microservices that are defined and orchestrated with Docker Compose ([7]; Fig. 1). The main application includes a Python-Flask [8] backend, a jQuery [9] frontend, and a uWSGI [10] application server that forwards requests to Flask from the NGINX reverse proxy server [11]. Flask, in turn, retrieves ASV and occurrence records from a PostgreSQL [12] database, turned into a RESTful API by the PostgREST [13] server. In addition, the main application delegates BLAST jobs to a worker, spawning additional worker processes when needed. Finally, the service configuration includes volumes for persistent storage of e.g. file uploads, BLAST and ASV database records.

## Results

The ASV portal provides options to submit and search for denoised metabarcoding data and associated occurrence records via intermediary ASV and BLAST databases (Fig. 1).

Data providers submit their data using a spreadsheet template based on the Darwin Core (DwC) standard for biodiversity data [14]. Specifically, the template corresponds to a DwC event core with associated contextual (‘extended Measurement or Facts’) and sequence-related (‘DNA derived data’ [15]) extensions. Each event is also associated with occurrences reported in ASV table format, i.e. as read counts given per sample (row) and ASV (column), rather than in the typical DwC occurrence format.

Submitted data files are curated and imported into the ASV database by portal administrators. A standard taxonomic annotation is then applied to each ASV, using current versions of selected classification algorithms and reference databases. The database schema also allows for successive re-annotations, enabling improved taxonomic accuracy and resolution as reference databases develop. Each DwC occurrence is, however, also assigned a unique taxon ID, based on the MD5 checksum of the underlying ASV sequence. This ensures that identification is consistent between data providers, and unaffected by changes in the mapping of ASVs to different taxon concepts.

Imported datasets are shared with GBIF and LA via the Integrated Publishing Toolkit [16]. The ASV database schema includes linked DwC views that can be accessed and filtered to create a new data resource in the IPT. The portal administrator then invites the data provider to fill in dataset-level metadata in the IPT form, before the dataset is formally published and made available to LA users.

**Fig. 2:**
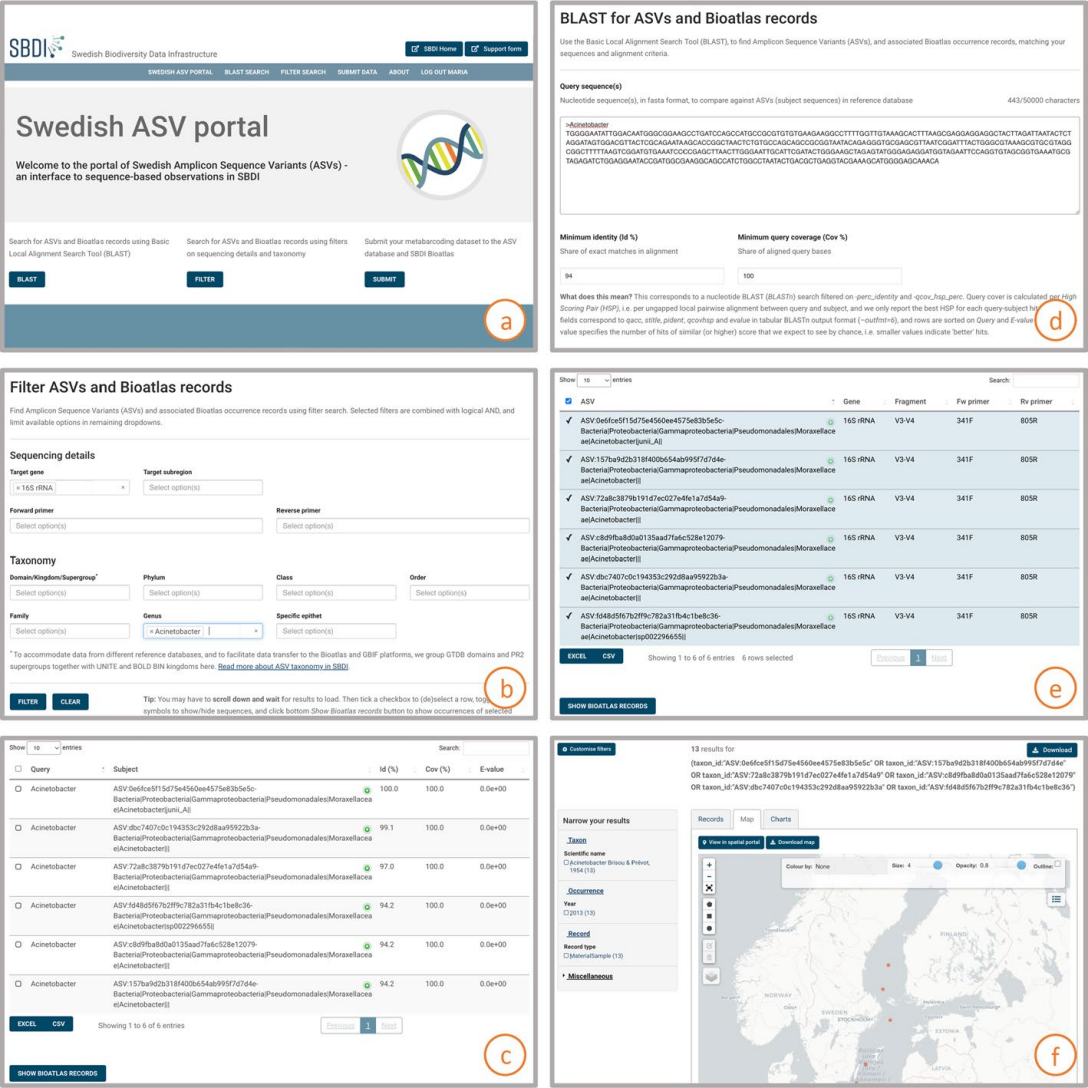
Use-case: Searching for *Acinetobacter* sequences and occurrence records in the ASV portal. A user interested in finding denoised sequences and associated occurrence records of a specific taxon, is presented with two search options in the start page of the ASV portal: BLAST and filter search (**a**). Filtering for ASVs derived from the 16S rRNA target gene in the genus *Acinetobacter* (**b**), produces a list of six ASVs, available for direct download (**c**). Alternatively, BLAST:ing against a known marker sequence from the targeted taxon (**d**), results in a corresponding list of ASVs (**e**). The user then opts for showing associated occurrence records in the main atlas platform (**f**), where data can be visualised and analysed together with other species observations as well as environmental and contextual data layers

The ASV portal provides two options for finding ASVs and published LA records: BLAST or FILTER search. In the BLAST form, users can paste in FASTA sequences, and set the minimum identity and query coverage of returned hits. Sequences are then aligned against a BLAST database that portal administrators rebuild when new data are imported into the ASV database. The FILTER form lets the user filter out ASVs based on sequencing details (e.g. target gene) and taxonomy. Search results are presented in similar, paginated tables in which users can select specific ASV records. Users can download these directly, in Excel or delimited text format, or choose to explore associated occurrence records in the LA platform. An illustrated use case for ASV portal search is given in Fig. 2, and a video tutorial covering both data submission and searching is available on YouTube [17].

### Future development

The ASV portal is currently an integral part of the Swedish LA instance [18], but given the rate at which sequence-based biodiversity data are being collected around the world, we envision that the LA community at large will benefit from our initiative to integrate this data source. We aim to keep the portal up to date, and welcome user requests, as well as contributions from biodiversity informatics programmers that want to join this open source project. The application will likely need to be optimised to support larger amounts of data in the future, and possible development includes adding an option for direct API access to data, by providing custom R and Python client libraries for this.

## Conclusion

The ASV portal is a Python-Flask web interface that integrates DNA sequence-based biodiversity data into the Living Atlas platform, where they can be combined with a multitude of other data sources to e.g. model processes that affect entire biotas, and to predict system-wide effects of environmental change. Most importantly, the portal provides straightforward options to submit data from metabarcoding studies in a convenient (ASV table) format, and to search for ASVs and associated occurrence records using sequence alignment (BLAST), as well as filters on e.g. target genes or primers. The application is developed in open collaboration, and containerized for easy deployment on any platform.

### Availability and requirements

*Project name*: ASV portal.

*Project home page*: https://asv-portal.biodiversitydata.se (running instance), https://github.com/biodiversitydata-se/mol-mod (development repository).

*Archived version*: https://zenodo.org/record/6394275.

*Operating systems*: Platform independent.

*Programming language*: Python, jQuery.

*Other requirements*: Docker and Docker Compose.

*License*: CC0 1.0 Universal (jQuery, DataTables and select2 components: MIT license).

*Any restrictions to use by non-academics*: None.

## Data Availability

The dataset supporting the conclusions of this article is available from the db-backup folder of the development repository (https://github.com/biodiversitydata-se/mol-mod) and the archived resource (https://zenodo.org/record/6394275). A video tutorial of the application is available on YouTube (https://www.youtube.com/watch?v=9P1qcJqZQtA).

## Abbreviations

API: Application programming interface
ASV: Amplicon sequence variant
BLAST: Basic local alignment search tool
DwC(A): Darwin core (archive)
eDNA: Environmental DNA
GBIF: Global biodiversity information facility
IPT: Integrated publishing toolkit
LA: Living Atlas
SBDI: Swedish biodiversity data infrastructure
